# Microwave Assisted Extraction of Phenolic Compounds from Four Different Spices

**DOI:** 10.3390/molecules15096365

**Published:** 2010-09-09

**Authors:** Monica Gallo, Rosalia Ferracane, Giulia Graziani, Alberto Ritieni, Vincenzo Fogliano

**Affiliations:** Department of Food Science, University of Naples “Federico II”, Via Università 100, Parco Gussone Ed.84, 80055, Portici, Naples, Italy

**Keywords:** spices, antioxidant activity, phenolic compounds, MAE, food composition

## Abstract

Spices and herbs are known not only for their taste, aroma and flavour, but also for their medical properties and value. Both spices and herbs have been used for centuries in traditional medical systems to cure various kinds of illnesses such as common cold, diabetes, cough and cancers. The aim of this work was the comparison between two different extractive techniques in order to get qualitative and quantitative data regarding bioactive compounds of four different spices (*Cinnamomum zeylanicum*, *Coriandrum sativum*, *Cuminum cyminum*, *Crocus sativus*). The plants were extracted employing ultrasonication and microwave-assisted extractions. The efficiency of extraction of bioactive compounds obtained with the microwave extraction process was in general about four times higher than that resulting from sonication extraction. The various extracts obtained were analyzed for their antioxidant activity using ABTS, DPPH and FRAP assays and for their total polyphenolic content. It can be concluded that microwave-assisted extractions provide significant advantages in terms of extraction efficiency and time savings.

## 1. Introduction

Spices and herbs have been investigated for their antioxidant properties for at least 50 years, in particular, spices are known to possess a variety of antioxidant effects and properties [1,2]. In recent decades, a number of phenolic substances were isolated from a variety of spice sources, including phenolic acids, flavonoids, phenolic diterpenes and volatile oils [3,4,5,6]. Phenolic compounds in these plant materials are closely associated with their antioxidant activity. They are also known to play an important role in stabilizing lipid peroxidation and to inhibit various types of oxidizing enzymes [7,8].

This antioxidant action make the diverse group of phenolic compounds an interesting target in the search for health-beneficial phytochemicals and also offer a possibility to use phenolic compounds or extracts rich in them in lipid-rich foods to extend shelf life [9], indeed the presence of antioxidative and antimicrobial phenolic constituents in many spices gives food-preserving properties [10,11].

Flavonoids and other plant phenolics, such as phenolic acids, stilbenes, tannins, lignans and lignin are especially common in leaves, flowering tissues and woody parts such as stems and barks [12]. They generally occur as glycosylated derivates in plants, although conjugation with inorganic sulfate or organic acids as well as malonylation are also known [13]. The antioxidant activity of phenolics is mainly due to their redox properties, which allow them to act as reducing agents, hydrogen donors and singlet oxygen quenchers. In addition, they have a metal chelation potential [14]. 

Several extraction techniques and solvents are used for obtaining antioxidant extracts from plant sources. Extraction techniques include solvent extraction (SE) [15], microwave assisted extraction (MAE) [16], soxhlet extraction and supercritical fluid extraction [17]. Among these, MAE is a relatively new method used for the extraction of natural products [18]. Pan *et al*., [19] had earlier shown that MAE was more effective than conventional extraction methods in the extraction of tea polyphenols and tea caffeine. Hong *et al*. used MAE to optimize the extraction of phenolic compounds from grape seeds [20].

A recent paper in the literature [21], reports that the best extraction of phytocomponents from flowering tops of *Melilotus officinalis* was obtained employing 50% aqueous ethanol either with microwave assisted extraction or with ultrasound assisted extraction. Waksmundzka-Hajnos *et al*., [22] studied the optimal conditions for the extraction of furanocoumarins from fruits of *Archangelica officinalis* and concluded that some compounds such as imperatorin and phellopterin may be transformed during pressurised microwave assisted extraction; therefore the MAE cannot be considered an advisable method for the furanocoumarin recovery.

Ganzler *et al*., [18] showed that a microwave-assisted extraction system for biologically active compounds has many advantages over other conventional extraction methods. Microwave-assisted extraction methods required shorter time, less solvents, provide higher extraction rates and better products with lower costs. Numerous biologically active compounds have been extracted by application of microwave-assisted extraction, such as extraction of taxanes from *Taxus brevifolia* needles [23], extraction of azadiractine related limonoids from *Azadirachta indica* seed kernels [24], extraction of glycyrrhizic acid from *Glycyrrhizia glaubra* root [25], extraction of tanshinones from *Salvia miltorrhiza bung* [26], extraction of artemisinin from *Artemisia annua* [27] and extraction of ginsenosides from *Panax ginseng* root [28]. 

The aim of this work was the comparison between two different extractive techniques in order to study the qualitative and quantitative data regarding bioactive compounds of four different spices (*Cinnamomum zeylanicum*, *Coriandrum sativum*, *Cuminum cyminum*, *Crocus sativus*). The plants were extracted employing ultrasonication and microwave-assisted extractions.

## 2. Results and Discussion

### 2.1. Total phenols and antioxidant activity

The optimised extraction conditions used in the MAE were determined after preliminary tests showing that severe conditions of microwave power and temperatures gave recoveries of polyphenol compounds about 30% lower than ultrasound assisted extraction. 

The analysis of total polyphenols (Folin) of different extracts obtained suggested that the microwave-assisted method was more effective compared to the ultrasound extraction method and it could be used as an effective method to extract antioxidant components considering factors such as the extraction time and the solvent wastage. In particular, results showed higher recoveries for *Cinnamomum zeylanicum* (three times higher), *Cuminum cyminum* (four times higher), *Crocus sativus* (six times higher), while for *Coriandrum sativum* no significative differences between the two methods have been found. Our data shown that the amount of total phenolics varied widely in spice samples analyzed and ranged from 41 to 2939 mg GAE/100 g dry material (Table 1) between two techniques. (Table 1). 

In hydroalcoholic conventional extracts of *Crocus sativus* total antioxidant concentration was of 500.21 mg/100 g on dry weight, this result was according with literature data which reported amount of polyphenolic compounds of 526.72 mg/100 g on dry weight [29].

For *Cuminum cyminum* the values reported in literature for total polyphenols in hydroalcoholic extracts were of 230 mg/100 g dry weight and were comparable to the values obtained in our ultasound assisted extracts that showed an amount of total phenols of 290.29 mg/100 g dry weight [30].

The total phenolic content for *Cinnamomum zeylanicum* was 507 mg/100 g on dry weight, with respect to 49 mg/100 g dry weight reported in literature [31] for barks. Our value was referred to leaves and was in accordance with the results showed by Prasad *et al*., regarding the flavonoid contents and antioxidant activities from *Cinnamomum* species [32].

*Coriandrum sativum* showed a value of 41.81 mg/100 g of dry weight, a concentration that was low if compared with values reported in literature [32,33], which amounted to 520 mg/100 g dry weight for the whole plant and 880 mg/100g dry weight for the leaves. On the other hand, the values reported in our study referred to seeds and not to entire plants or leaves as in literature. 

The four species were rich in phenolic constituents and demonstrated good antioxidant activity measured by different methods. The antioxidant activity was carried out using ABTS [37], DPPH [38] and FRAP assays [39], which determined the disappearance of free radical solutions using a spectrophotometer. 

The antioxidant activity evaluated with the ABTS test allowed us to obtain the results reported in Table 2. In particular, *Cinnamomum zeylanicum, Cuminum cyminum* and *Crocus sativus* microwave-assisted extracts have a strong antioxidant activity compared to ultrasound assisted extracts, while for *Coriandrum sativum* the conventional extraction allows to obtain more effective extracts in terms of antioxidant capacity.

Among the different matrices analyzed, *Cinnamomum zeylanicum*, extracted with microwaves shows the greatest antioxidant activity measured by the ABTS assay (3.17 mmol Trolox^®^/100 g), while the seeds of *Coriandrum sativum*, extracted with microwave, are those who have the lowest antioxidant activity (0.035 mmol Trolox^®^/100 g). On the other hand, microwave-assisted extract of *Crocus sativus*, showed an antioxidant effectiveness 28 times higher than the extract obtained with ultrasound assisted extraction considering the ABTS assay. In the literature 1,243 μmol Trolox®/100g of dry weight and 1,064 μmol/100 g of dry weight were reported for the acetonic and methanolic extracts of *Cinnamomum zeylanicum*, respectively [33]. As shown in Table 2 the values obtained in the ultrasound assisted extracts are in accordance with the literature data.

In the case of DPPH, the values of antioxidant activity of *Cinnamomum zeylanicum*, *Cuminum cyminum* and *Crocus sativus* extracts obtained from the two extractive techniques are comparable, while for *Coriandrum sativum* the antioxidant activity of ultrasound assisted extract was almost three times greater than the microwave assisted extract (Table 2). This result could be due to different affinities of *Coriandrum sativum* antioxidant compounds presents in ultrasound assisted extract towards DPPH radical species. In literature, Politeo *et al*. [34] reported an antioxidant activity value of 57.75 measured with the DPPH assay expressed as an inhibition percentage for *Coriandrum sativum* extracts, while Tomaino *et al*. [35] reported in *Cinnamomum zeylanicum * extracts a percentage of inhibition of 55.30. Our extracts obtained with ultrasound assisted process have an antioxidant activity expressed as inhibition percentage of 74.37 in *Coriandrum sativum* and 90.45 in *Cinnamomum zeylanicum,* therefore our results show high inhibition percentage values, probably due to the diversity of origin and/or cultivars of matrix analyzed.

The data obtained with FRAP assay are represented in Table 2. All spices analyzed showed an antioxidant activity highest for the microwave-assisted extraction. Among the samples analyzed, the microwave-assisted extract of *Crocus sativus* showed the highest antioxidant activity (391 mmol Trolox®/100 g), while *Coriandrum sativum* had the lowest antioxidant activity (68 mmol Trolox®/100 g).

The results allow us to claim that the microwave-assisted extraction is a particularly advantageous technique especially for *Crocus sativus*, which had an antioxidant activity about 45 times higher than ultrasound assisted extract. In the literature it was reported that *Crocus sativus* extract showed a total antioxidant activity calculated as sum of hydrophilic and lipophilic antioxidant activity of about 73.94 mmol of Fe^2+^/100 g dry weight [36]. Our results obtained for ultrasound extracts showed an antioxidant activity expressed as mmol of Trolox®/100g of dry matter of 8.45; this result allow to assert that *Crocus sativus* was the matrix best extracted using microwaves, probably for its typical structure that enables an advantageous recovery of bioactive metabolites. This result could be attributed to the higher dielectric susceptibility of the matrix and to the better interaction between solvent and the *Crocus sativus* in MAE conditions. Moreover, crocetins are water soluble compounds esterified with one or two sugar moieties, therefore they easily adsorb microwave energy. 

## 3. Materials and Methods 

### 3.1. Chemicals

All reagents and solvents HPLC grade were purchased from Merck (Darmstadt, Germany). The spices (*Cinnamomum zeylanicum*, *Coriandrum sativum*, *Cuminum cyminum*, *Crocus sativus*) were bought in a local market from a specialized retailer of spices for human consumption. Each sample was contained in a small glass bottle sealed with a plastic screw cap. The products, harvested in 2008, were in different forms: minced desiccated leaf samples: *Cinnamomum zeylanicum*; powdered samples: *Crocus sativus* stigma; samples of seeds: *Coriandrum sativum* and *Cuminum cyminum*. 

(*S*)-(-)-6-Hydroxy-2,5,7,8-tetramethylchroman-2-carboxylic acid (Trolox^®^), 2,2’-azino-bis(3-ethyl-benzothiazoline-6’-sulfonic acid) diammonium salt (ABTS), gallic acid, potassium persulfate, 2,2-diphenyl-1-picrylhydrazyl (DPPH), ferric chloride, Folin Ciocalteau, anhydrous sodium acetate, reagent and 2,4,6-tri(2-pyridyl-*s*-triazine) (TPTZ), were purchased from Sigma (Milan, Italy), as were caffeic acid, cinnammic acid, chlorogenic acid, quercetin, luteolin, apigenin, quercetin-rhamnoglucoside (rutin), luteolin glucoside and apigenin glucoside standards. 

### 3.2. Microwave-assisted extraction (MAE)

An ETHOS 1 microwave-oven (Milestone, Shelton, CT, USA) equipped with 10 TFM Teflon closed vessels and a ATC-400FO Automatic Fiber Optic temperature control system was used for microwave assisted extractions (MAE). Extractions were performed at 200 W and at 50 ºC, a magnetic stirring rod was added in each vessel. The spices were extracted using the following method: sample (1 g) was extracted with ethanol/water (50:50 *v*/*v*, 20 mL), the extractions being carried at 200 W using magnetic stirring at 50% of nominal power and a temperature of 50 ºC for 18 min. All samples were filtered through a 0.45 μm nylon syringe filter (Millipore) before chromatographic and antioxidant analysis. All extractions were performed in triplicate.

### 3.3. Ultrasound assisted extraction (UAE)

Sample (3g) was extracted with ethanol/water (50:50 v/v, 30 mL), the extractions were performed in a ultrasonic bath (Astrason the Heat System (Germany) with a working frequency of 33 KHz. The samples after sonication at room temperature for 30 min, were centrifuged to 4,000 rpm, at 4 ºC, filtered with filter paper and analyzed by mass spectrometry. The ultrasonic plant material extraction procedure was repeated three times.

### 3.4. Antioxidant activity: ABTS assay

Antioxidant capacity assay was performed using an UV-VIS recording spectrophotometer (Shimadzu, Japan) by the improved ABTS^. +^ method as described by Re *et al*. [37]. ABTS^. +^ radical cation was generated by reacting 7 mM ABTS and 2.45 mM potassium persulfate after incubation at room temperature (23 ºC) in dark for 16 h. The ABTS^. +^ solution was diluted with ethanol to an absorbance of 0.700 ± 0.050 at 734 nm. The filtered sample was diluted with 70% methanol so as to give 20–80% inhibition of the blank absorbance with 0.1 mL of sample. ABTS^. +^ solution (1 mL, with absorbance of 0.700 ± 0.050) was added to the tested samples (0.1 mL) and mixed thoroughly. The reactive mixture was allowed to stand at room temperature for 2.5 min and the absorbance was immediately recorded at 734 nm. Trolox^®^ standard solution (final concentration 0–15 μM) in methanol was prepared and assayed at the same conditions. The absorbance of the resulting oxidized solution was compared to that of the calibrated Trolox^®^ standard. Results were expressed in terms of Trolox^®^ equivalent antioxidant capacity (TEAC, mmol Trolox^®^ equivalents per 100 g dry weight of plant).

### 3.5. Antioxidant activity: DPPH assay

The DPPH radical-scavenging activity was determined using the method proposed by Yen and Chen [38]. DPPH (100 μM) was dissolved in pure ethanol (96%). The radical stock solution was prepared fresh daily. The DPPH solution (1 mL) was added to the polyphenol extract (1 mL) with ethanol (3 mL). The mixture was shaken vigorously and allowed to stand at room temperature in the dark for 10 min. The decrease in absorbance of the resulting solution was monitored at 517 nm at 10 min. The results were corrected for dilution and expressed in μM Trolox^®^ per 100 g dry weight (dw). All determinations were performed in triplicate.

### 3.6. Antioxidant activity: FRAP assay

This assay was based on the reducing power of antioxidant compouds toward ferric salt [39]. Antioxidants reduce the ferric ion (Fe^3+^) to the ferrous ion (Fe^2+^) and the latter forms was a blue complex (Fe^2+^/TPTZ), which increases the absorption at 593 nm. Briefly, the FRAP reagent was prepared by mixing acetate buffer (300 μM, pH 3.6), a solution of 10 μM TPTZ in 40 μM HCl, and 20 μM FeCl_3_ at 10:1:1 (v/v/v). The reagent (300 μL) and sample solutions (10 μL) were added to each well and mixed thoroughly. The absorbance was taken at 593 nm after 10 min. Standard curve was prepared using different concentrations of Trolox^®^. All solutions were used on the day of preparation. The results were corrected for dilution and expressed in μM Trolox^®^ per 100 g dry weight (dw). All determinations were performed in triplicate.

### 3.7. Determination of total phenolics (Folin-Ciocalteu)

Total polyphenol content was measured using the Folin-Ciocalteu colorimetric method described previously by Gao **et al*.* [40]. Spice extracts (100 μL) were mixed with Folin-Ciocalteu reagent (0.2 mL) and H_2_O (2 mL), and incubated at room temperature for 3 min. Following the addition of 20% sodium carbonate (1 mL) to the mixture, total polyphenols were determined after 1 h of incubation at room temperature. The absorbance of the resulting blue colour was measured at 765 nm with a UV-VIS spectrophotometer. Quantification was done with respect to the standard curve of gallic acid. The results were expressed as gallic acid equivalents (GAE), milligrams per 100 g of dry weight (dw). All determinations were performed in triplicate (*n* = 3).

## 4. Conclusions

In this study two different methods for the bioactive metabolites extraction (ultrasound and microwave assisted extraction) were compared. 

The analysis of total polyphenols (Folin) of various extracts obtained suggested that the microwave-assisted method was more convenient compared to the ultrasound extraction method especially for *Cinnamomum zeylanicum*, *Cuminum cyminum*, *Crocus sativus*, while for *Coriandrum sativum* no significative differences between the two methods have been found.

The antioxidant activity measured with ABTS, DPPH and FRAP assays activity highlighted that the extracts obtained using microwaves were richer in antioxidant metabolites than those obtained by ultrasonic extraction, being a good source of antioxidants.

The use of MAE in analytical laboratories should increase in the next few years, especially thanks to the reasonable cost of the equipment, suggesting that this field of application should expand in the near future. 

## Figures and Tables

**Table 1:** Total phenolic contents of spice extracts obtained with ultrasound and microwave assisted extraction.

Sample	Total phenolic content (mg gallic acid/100 g)	
	UAE	MAE
*Coriandrum sativum*	41,812 ± 2,765	82,091 ± 8,432
*Cinnamomum zeylanicum*	506,597 ± 23,518	1679,201 ± 65,333
*Cuminum cyminum *	290,296 ± 13,545	1159,542 ± 21,239
*Crocus sativus*	500,213 ± 34,745	2939,472 ± 24,610

**Table 2:** Antioxidant activity of spice extracts obtained with ultrasound and microwave assisted extraction.

Sample	Antioxidant activity (ABTS) mmol Trolox^®^/100 g		Antioxidant activity (DPPH) % inhibition		Antioxidant activity (FRAP) mmol Trolox^®^/100 g	
	UAE	MAE	UAE	MAE	UAE	MAE
*Coriandrum sativum*	0,080	0,035	74,379	25,565	1,198	68,765
*Cinnamomum zeylanicum*	1,271	3,172	90,451	91,789	8,176	240,045
*Cuminum cyminum *	0,217	2,671	85,432	88,432	50,345	140,319
*Crocus sativus*	0,045	1,258	15,692	19,673	8,450	391,123
